# Reliability Evaluation Based on Mathematical Degradation Model for Vacuum Packaged MEMS Sensor

**DOI:** 10.3390/mi13101713

**Published:** 2022-10-11

**Authors:** Guizhen Du, Xianshan Dong, Xinglong Huang, Wei Su, Peng Zhang

**Affiliations:** 1 Institute of Advanced Wear & Corrosion Resistance and Functional Materials, Jinan University, Guangzhou 510632, China; 2 Science and Technology on Reliability Physics and Application Technology of Electronic Component Laboratory, China Electronic Product Reliability and Environmental Testing Research Institute, Guangzhou 511370, China; 3 College of Mechanical and Electrical Engineering, Central South University, Changsha 410083, China

**Keywords:** MEMS sensor, reliability evaluation, vacuum degradation, mathematical model

## Abstract

Vacuum packaging is used extensively in MEMS sensors for improving performance. However, the vacuum in the MEMS chamber gradually degenerates over time, which adversely affects the long-term performance of the MEMS sensor. A mathematical model for vacuum degradation is presented in this article for evaluating the degradation of vacuum packaged MEMS sensors, and a temperature-accelerated test of MEMS gyroscope with different vacuums is performed. A mathematical degradation model is developed to fit the parameters of the degradation of Q-factor over time at three different temperatures. The results indicate that the outgassing rate at 85 °C is the highest, which is 0.0531 cm^2^/s; the outgassing rate at 105 °C is the lowest, which is 0.0109 cm^2^/s; and the outgassing rate at 125 °C is in the middle, which is 0.0373 cm^2^/s. Due to the different mechanisms by which gas was released, the rate of degradation did not follow this rule. It will also be possible to predict the long-term reliability of vacuum packaged MEMS sensors at room temperature based on this model.

## 1. Introduction

The micro-electro-mechanical system (MEMS) is a micro-intelligent system utilizing microelectronics, micromechanics, and other technologies to integrate sensors, actuators, and signal transmission units [1]. Micro-electro-mechanical sensors possess the characteristics of small volume, light weight, and low cost, and are on the verge of achieving passivity, miniaturization, and anti-interference capabilities [2]. As technology advances, MEMS sensors have become widely used in a variety of fields, including consumer electronics, aerospace, military equipment, biomedicine, and others [3,4,5,6]. It is common for MEMS sensors to have movable structures, such as gyroscopes, accelerometers, and filters that are based on resonant structures. Vacuum packaging can reduce the air damping of their movable structures, improving their performance. In spite of this, the vacuum in the MEMS chamber will gradually degenerate over time, affecting directly the performance of vacuum packaged MEMS sensors, while seriously affecting their long-term reliability. Vacuum degradation and failure are common problems associated with vacuum-packaged MEMS sensors. As a consequence, the study of the vacuum degradation and long-term reliability of vacuum packaged MEMS sensor is of great importance for the development and application of vacuum packaged MEMS sensors.

In the present day, some scholars have conducted in-depth research into the vacuum degradation of MEMS devices. Through vacuum reflow technology, Liang et al. [7] were able to ensure good sealing of the packaging cavity and placed getters within the cavity to maintain a stable vacuum inside the cavity. As a result of testing the resonant gyroscope for nine months, Wu et al. [8] prepared multilayer getters, which were activated at 300 °C, and the getters showed effective performance. As reported by Tepolt et al. [9], the initial outgassing of the material in the packaging cavity, as well as the vacuum pressure required to stabilize the devices, was measured. In order to optimize the processing technology and minimize the exhaust amount of the MEMS devices cavity without serious degradation, the data was compared with the specific getter capacity data. A mathematical model has been developed by Lu et al. [10] that provides a theoretical basis for directly comparing the behavior of different gases entering the sealing cavity. According to the principles of thermodynamics and hydrodynamics, He et al. [11] determined the relationship between Q-factor, air pressure, and gas number of MEMS sensor cavities. For MEMS resonators, Candler proposed a single-chip packaging method [12]. Using thick polysilicon packaging, the MEMS resonator was packaged under a pressure of less than 1 Pa, and the reliability test was conducted under high temperatures. As a result, the majority of research focuses on improving the packaging process and developing packaging materials for MEMS devices. Some researchers devote a great deal of time to assessing the long-term reliability of vacuum-packaged sensors. Currently, there are few mature theoretical models of the effect of deflation of the internal materials of MEMS devices on the long-term reliability of these devices. 

As part of this paper, an evaluation of the reliability of vacuum-packaged MEMS sensors is presented. In order to analyze the degradation failure of vacuum packaged MEMS sensors, a degradation failure model was developed. Additionally, the parameters of the model were obtained by fitting data obtained from MEMS sensors with varying vacuum degrees at multiple temperature points. According to the results, the model is well suited to the experimental data. Further, the model was used to predict the vacuum degradation of a vacuum-packaged MEMS sensor at room temperature based on the model obtained.

## 2. Materials and Methods

### 2.1. Model of Vacuum Degradation

In vacuum packaged MEMS sensors, vacuum degree is degraded mainly due to the release of gas inside the cavity [13], including gas molecules adsorbed on the inner wall of the cavity, gas molecules in the material inside the cavity, etc. High temperatures are the main source of sensitive stress [14]. It is generally true that vacuum-packaged MEMS sensors have resonant structures, which can be used as a measure of the level of vacuum inside the cavity of the package [15]. Hence, this paper first establishes a mathematical model for predicting the degradation of the Q-factor of vacuum packaged MEMS sensors when exposed to high temperatures. Here is a description of how the mathematical model is constructed.

Material outgassing from vacuum-packaged MEMS sensors is considered an unsteady process, and the diffusion coefficient in different directions is regarded as a constant. Based on Fick’s second law:

(1) $$\frac{\partial_{n}}{\partial_{t}}=D\nabla^{2}n$$

In this formula, *n* represents the number of gas molecules per unit cross-sectional area, *t* represents the diffusion time, and *D* represents the diffusion coefficient. The Gilliland formula can be used to express the diffusion coefficient *D* as follows:

(2) $$D=\frac{435.7\sqrt{2}\sqrt[{}]{T^{3}}}{2p\sqrt{\mu }\sqrt[{3}]{V}}$$

where *T* denotes the thermodynamic temperature, *p* is the total pressure, *μ* is the molecular weight, and *V* is the liquid gram molar volume of the gas at its normal boiling point.

After vacuum packaging, the MEMS sensor cannot maintain an absolute vacuum state in its cavity, so there will be a certain amount of gas in the initial state, which is directly related to the initial Q-factor of the samples. Considering this, assuming there are *N*_0_ gas molecules in the sealed cavity at the beginning, and *N*_tot_ is the total number of gas molecules released, the boundary condition for the number of molecules inside the cavity *N*(*t*) over time is:

(3) $$N\left(t\right)=\left\{\begin{array}{c} N_{0},t=0\\ N_{0}+N_{\mathrm{tot}},t=\infty \end{array}\right.$$

Based on the above conditions, the Fourier series method may be used to calculate the diffusion equation as follows:

(4) $$n\left(z,t\right)=\frac{N_{\mathrm{tot}}}{V}+\overset{\infty }{\underset{i=1}{\sum }}\frac{2N_{\mathrm{tot}}}{V}\times e^{-\lambda_{i}Dt}\times \cos\left(\frac{i\pi z}{l_{z}}\right)$$

As a result, the number of free gas molecules can be expressed as follows:

(5) $$\begin{array}{c} \frac{1}{Q\left(t\right)}=A\cdot N\left(t\right)=A\cdot \left[N_{0}+N_{\mathrm{tot}}\left(t\right)\right]=A\cdot \left[\frac{A}{Q_{0}}+N_{\mathrm{tot}}\cdot \left(1-e^{-D_{T}\cdot t}\right)\right]\\ =\left(\frac{1}{Q_{0}}+A\cdot N_{\mathrm{tot}}\right)-A\cdot N_{\mathrm{tot}}\cdot e^{-D_{T}\cdot t} \end{array}$$

MEMS sensors that are vacuum packaged can be described by the following vacuum degradation model:

(6) $$p\left(t\right)\propto \frac{1}{Q\left(t\right)}\propto N\left(t\right)=a-b\times e^{-c\times t}$$

In the formula, *a* = 1/*Q*_0_ + A × *N*_tot_, *b* = A × *N*_tot_, *c* = *D_T_*, and the physical meanings of model parameters *a*, *b*, and *c* are as follows:

Parameter *a* represents the final total number of gases inside the sample cavity, which determines the final Q-factor of the sample. Depending on parameter *b*, the degree of degradation of the sample is determined by the number of gases released inside the sample cavity. Parameter *c* characterizes the degradation speed of the sample, which is related to temperature.

With time, the vacuum degree of the vacuum-packaged MEMS sensor decays exponentially. By fitting the experimental data to the above-mentioned model, parameters *a*, *b*, and *c* can be obtained. The above analysis also reveals that 1/(*a−b*) is the initial value of the Q-factor of the sample.

### 2.2. Experiment of Reliability Evaluation

#### 2.2.1. Sample

As it is difficult to detect and activate the MEMS microstructure of the MEMS products electrically, we customized a batch of samples. It is typical to find MEMS gyroscopes in commercial applications. The structure of the device must be in a resonant state, and it is usually packaged in a vacuum. In order to investigate vacuum degradation of vacuum packaged MEMS devices, this paper selects the sensitive structure of a MEMS gyroscope as the subject of experimental research.

**Figure 1:**
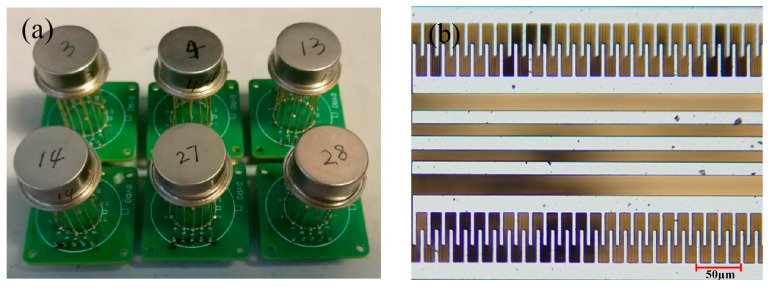
Schematic illustration of (**a**) test sample and (**b**) internal structure diagram.

As shown in Figure 1b, MEMS gyroscopes have comb structures as their internal structure. During the operation of the gyroscope, the position of the combs will change, as will the capacitance between them. It is possible to determine the value based on the change in capacitance. As a result, the vacuum encapsulated gyroscope will have difficulty moving the comb, which will adversely affect the gyroscope’s performance.

As shown in Figure 1a, three types of device-level packaged MEMS gyroscopes were customized with different vacuum degrees.

Table 1 provides information regarding the vacuum packaging of the samples. There were a total of 18 samples, which were divided into three groups. Samples that were highly vacuum packaged were H1 and H6, medium vacuum packaged samples were M1 and M6, and low vacuum packaged samples were L1 and L6.

#### 2.2.2. Test Method and System

**Figure 2:**
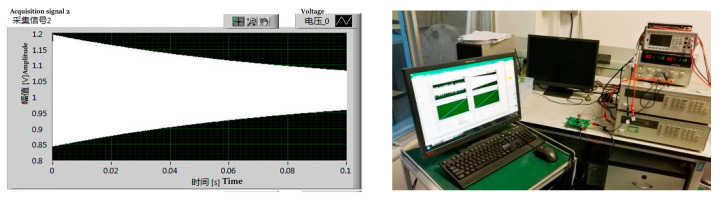
Q-factor test frequency response diagram and test system.

Considering that the Q-factor can be used to characterize the vacuum of a MEMS cavity, the Q-factor of our sample was evaluated. As shown in Figure 2, we developed a Q-factor test method based on transient excitation [16] for the above-mentioned test samples with different levels of vacuum.

Q value measurement is based on the oscillating principle [17]. Labview software, an NI PXI 4461 acquisition card, an analog circuit, and a MEMS sensor are included in the test system. A part of the test process is as follows: by applying excitation signals to the resonant structure of the MEMS sensor using the acquisition card, Labview software causes the sensor to enter the steady state. After stopping the excitation, the sensor is in a state of damped vibration. As a final step, the acquisition card collects the transient response voltage signal and passes it to Labview for analysis and processing. Using Labview software, it is possible to determine the resonant frequency and Q value rapidly by using wave peak detection technology, the cycle average method, and exponential fitting algorithm.

#### 2.2.3. Experiment

The environment has a significant impact on the reliability of vacuum-packaged MEMS sensors. It has been reported that the Q-factor of vacuum packaged MEMS gyroscopes will degrade with time at room temperature [13]. Based on theory and analysis, it has been tentatively determined that temperature affects the outgassing of internal materials.

**Figure 3:**
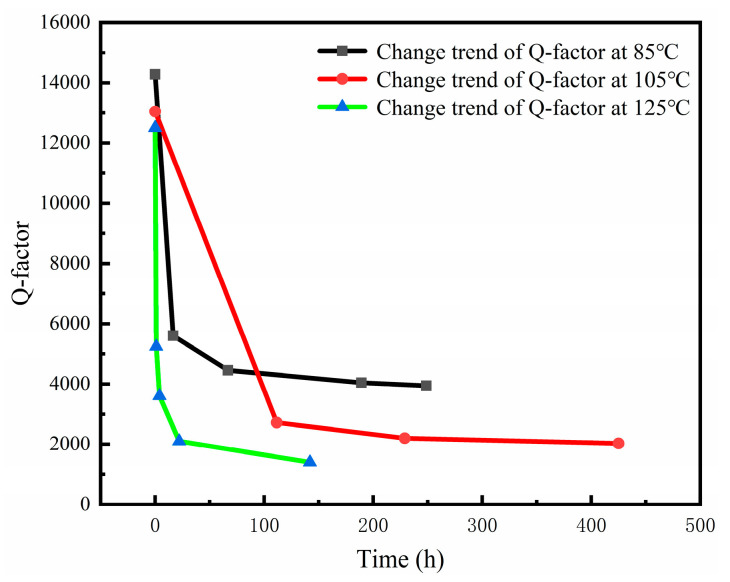
Degradation of Q-factor of vacuum packaged gyroscope with time at high temperature.

At the beginning of the research, a vacuum packaged MEMS gyroscope was baked at three different high temperatures and the Q-factor was monitored over time. According to Figure 3, the Q-factor of the MEMS gyroscope dropped sharply at high temperatures. Accordingly, the analysis of the theoretical and experimental results indicates that high temperature is the main stress contributing to the degree of vacuum degradation of MEMS sensors packaged in a vacuum.

At different temperatures, we conducted accelerated degradation experiments on the samples using the high-temperature accelerated test method based on MIL-STD-883. As shown in Table 2, samples with high vacuum, medium vacuum, and low vacuum were tested at three temperatures of 85 °C, 105 °C, and 125 °C. 

In order to conduct a reliability test, the following steps must be followed:

(1) Prior to the test, the Q-factor of the samples is measured at room temperature;

(2) Incubator temperature is raised to the specified temperature, and samples are placed in the incubator after the temperature reaches the specified temperature;

(3) The samples are baked at a high temperature, and then taken out of the incubator after *t*_1_ min;

(4) The Q-factor of the samples is determined after they have stood at room temperature for two hours;

(5) After the measurements have been completed, the samples are placed back into the incubator;

(6) Repeat steps (3) and (4) until the test is complete.

## 3. Results

### 3.1. Test Results

In Table 3, Table 4 and Table 5, reliability test results of samples at 85 °C, 105 °C, and 125 °C under high-temperature stress are presented.

**Figure 4:**
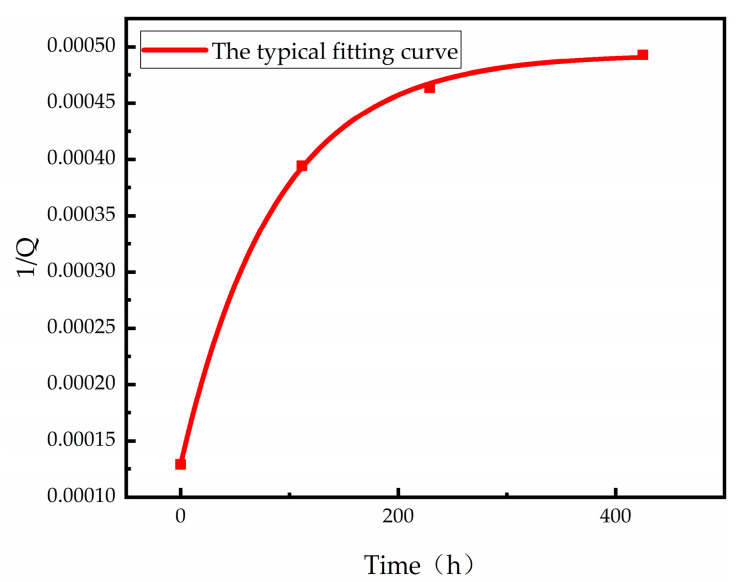
Q-factor degradation fitting curve of M4 sample at 105 °C.

As a result of the reliability test, we used the mathematical model presented in this paper for fitting. As can be seen from Figure 4, the typical fitting curve reaches a fitting degree of 1.00, indicating that the mathematical model established in this paper is more capable of reflecting the changes in vacuum degree over time under a high-temperature environment.

Having analyzed the data obtained from the experiment, the fitting parameters for the test samples with different vacuum degrees at three temperature points are determined, as shown in Table 6, Table 7 and Table 8.

The curve fitting degree for 18 samples exceeds 1.00 or is close to it, which confirms the accuracy of our mathematical model. As a result, it can also be concluded that the established model can accurately predict the degree of vacuum degradation of MEMS sensors that are vacuum packaged.

Based on the physical meaning of the fitting parameters, this paper analyses and discusses the obtained parameter values.

Parameter *a* specifies the total number of gases inside the sample cavity and determines the final Q-factor of the sample. According to the parameter values above for the fitting curve parameters, there are 18 samples at three different temperature points, and there is no significant difference between the *a* values of the high vacuum and medium vacuum samples at the same temperature point. This is due to the fact that the amount of gas released from high and medium vacuum samples is significantly greater than the amount of gas released from the initial sample. Compared to the high and medium vacuum samples at the same temperature, the low vacuum sample has a higher *a* value. Since the initial gas number of the low vacuum samples is higher, it is comparable with the total amount of gas released.

In addition, parameter *b* determines the degree of sample degradation by characterizing the total number of gases released inside the sample cavity. At the same temperature, the outgassing amount represented by the *b* value is essentially the same, which means that it has nothing to do with the vacuum degree of the sample, but only with its temperature. The higher the temperature, the greater the amount of outgassing.

Again, *c* characterizes the degradation speed of the samples, that is, the outgassing rate inside the sample, which is related to temperature. In high, medium, and low vacuums, the outgassing rates of samples remain essentially the same, indicating this parameter is not related to the sample’s vacuum degree, but only to its temperature.

### 3.2. Data Analysis

Parameters *b* and *c* of the six samples were relatively consistent at every temperature point, and the average value was taken as the value of parameter *b* and parameter *c* and summarized in Table 9.

Parameter *b* characterizes the total amount of gas released inside the sample, and the total amount of released gas inside the sealed cavity is related to the temperature.

According to the relationship between the total amount of gas out of non-metallic materials and temperature [18]:

(7) $$b=b_{0}\times e^{-B\times T}$$

The above equation can be transformed into a linear equation:

(8) $$\mathrm{In}b=\mathrm{In}b_{0}-B\times T$$

The data of parameter *b* and temperature *T* at three temperature points are shown in Table 10.

**Figure 5:**
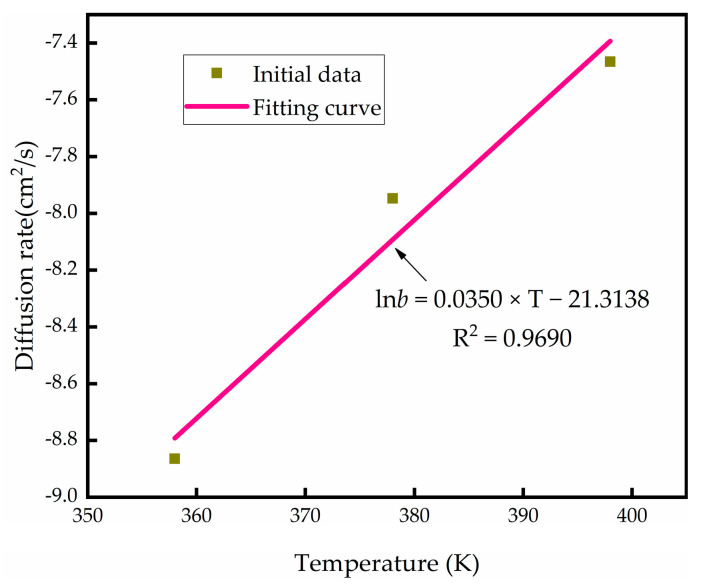
Linear fitting curve of *T* and ln*b.*

Taking *T* as the abscissa and In*b* as the ordinate, the linear fitting curve is shown in Figure 5, and the fitting equation is y=0.0350×x−21.3138.

The following parameters can be calculated from the equation of total gas output with temperature:

(9) $$\ln b_{0}=-21.3138;\;B=0.0350$$

Total outgassing volume and temperature are related as follows:

(10) $$b=e^{-21.3138+0.0350\times T}$$

**Figure 6:**
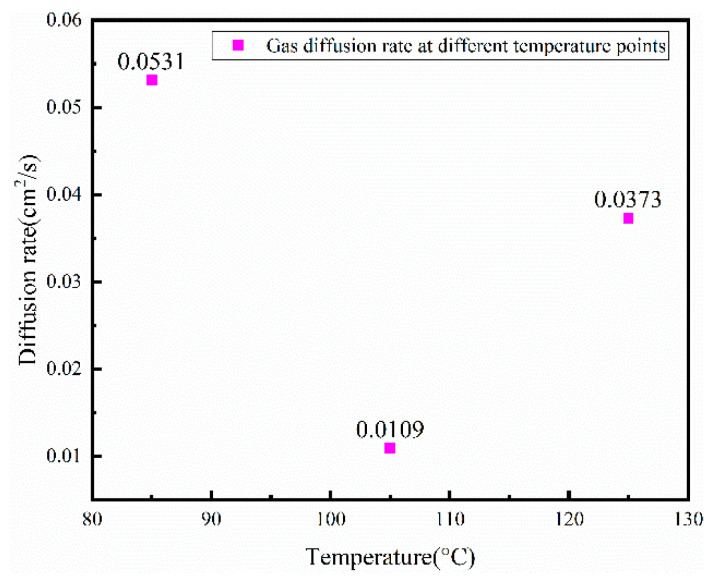
Outgassing rate at different temperature points.

In a sealed cavity, the rate of gas release is determined by parameter *c*, and the rate of gas release is related to the temperature inside the sealed cavity. According to theory, the higher the temperature, the greater the rate of outgassing. Figure 6 illustrates the results of the tests conducted in this paper. The outgassing rate at 85 °C is the highest, which is 0.0531; the outgassing rate at 105 °C is the lowest, which is 0.0109; and the outgassing rate at 125 °C is in the middle, which is 0.0373.

As a result of the experiments conducted, this paper concludes that the outgassing rate of the gas inside the sample is equal to the desorption rate of the gas minus the adsorption rate of the gas. It is important to note that the maximum outgassing rate occurs at 85 °C as a result of the desorption of physically adsorbed gas atoms or molecules from the surface of the material, whereas a higher temperature is required for gas in the deeper part of the material. The collision frequency between gas molecules or atoms increases at 105 °C, which increases the adsorption rate. As a result, the outgassing rate decreases at 105 °C. Gas atoms and molecules are desorbed at 125 °C by physical adsorption and chemical adsorption, resulting in an increase in the rate at which they are outgassed.

### 3.3. Reliability Prediction

Thus, we can obtain a mathematical model for describing the vacuum degradation of MEMS sensors as follows:

(11) $$p\left(t\right)\propto \frac{1}{Q\left(t\right)}\propto N\left(t\right)=a+\left(e^{-21.3138+0.0350\times T}\right)\times \left(e^{-c\times t}\right)$$

The majority of vacuum packaged MEMS gyroscopes and other MEMS sensors are used at room temperature, and the obtained mathematical model is used to predict the long-term reliability of MEMS gyroscopes under 25 °C vacuum degradation conditions.

The following parameters can be determined at a room temperature of 25 °C using the formula deduced above:

(12) $$b=e^{-21.3138+0.0350\times 298}=0.00001876$$

Using the previous analysis, it can be determined that 1/(*a*−*b*) can obtain the initial value of the Q-factor of samples, and we also know the value of *b*. Therefore, parameter *a*, which represents the final gas volume of the cavity, determines the final value of the MEMS sensor. Since the value of *c* is uncertain, *t* = ∞ is taken.

Table 11 shows the changes in Q-factor during storage (T = 25 °C) with time for different initial Q-factor.

## 4. Discussion

According to the established mathematical degradation model of vacuum-packaged MEMS sensors, the long-term reliability of vacuum-packaged MEMS sensors at room temperatures (25 °C) was predicted. The data are shown in Table 11, the vacuum degree of samples with different initial Q-factor degrades continuously over time; whereas samples with small initial Q-factor do not exhibit obvious degradation trends, samples with larger initial Q-factors demonstrate more obvious degradation trends. Furthermore, the degradation degree of the Q-factor of all samples tends to be flat with the increasing storage time.

At room temperature, the gas molecules adsorbed on the surface material of the sensor cavity are continuously released, which leads to the decrease of the vacuum degree of the sensor, thus reducing the reliability of the sensor. In the process of high-temperature accelerated aging, physical and chemical desorption of gas will occur, especially the gas released by some polymers in the cavity, which has a great impact on the reduction of vacuum degree.

## 5. Conclusions

In this paper, a theoretical model for the prediction of vacuum degradation of vacuum packaged MEMS sensors is established. Furthermore, combination with the experiments, the MEMS gyroscopes with high vacuum degree, medium vacuum degree, and low vacuum degree are used as examples to study the vacuum degradation law at different high temperatures and get the parameters of the model. The reliability life prediction of the vacuum packaged MEMS sensor under a room temperature environment (25 °C) is also carried out. The results show that our theoretical model can achieve a good prediction of vacuum degradation of vacuum packaged MEMS sensors. This is of great significance for improving the reliability of vacuum-packaged MEMS sensors and promoting the commercial application of vacuum-packaged MEMS sensors. In the next step, we will conduct further research on the variation law of the outgassing rate in the cavity with temperature, such as using a lower temperature test to obtain the relationship between the outgassing rate and temperature and predict the variation of the vacuum degree with time.

## Figures and Tables

**Table 1:** Design values of package air pressure and Q-factor for three kinds of samples.

Sample Serial Number	Package Air Pressure	Q-Factor
H1~H6	0.5 × 10^−^^3^ Torr	20,000
M1~M6	1.5 Torr	8000
L1~L6	20 Torr	1700

**Table 2:** Test conditions and grouping.

Environmental Stress	Samples	Test Parameter
85 °C	H1, H2, M1, M2, L1, L2	Q-factor
105 °C	H3, H4, M3, M4, L3, L4	
125 °C	H5, H6, M5, M6, L5, L6	

**Table 3:** Changes of Q-factor of different samples under high-temperature baking at 85 °C.

Time	H1	H2	M1	M2	L1	L2
0 h	14,281	19,390	7572	7822	2118	2018
16.50 h	5603	7578	4407	4563	1816	1766
66.75 h	4457	5914	3735	3856	1706	1673
189.25 h	4042	5163	3496	3579	1659	1624
248.75 h	3944	5034	3478	3550	1654	1621

**Table 4:** Changes of Q-factor of different samples under high-temperature baking at 105 °C.

Time	H3	H4	M3	M4	L3	L4
0 h	13,048	16,388	7710	7744	2072	2061
111.5 h	2721	3200	2572	2535	1439	1407
229 h	2201	2591	2202	2157	1320	1292
425 h	2030	2403	2076	2029	1265	1242

**Table 5:** Changes of Q-factor of different samples under high-temperature baking at 125 °C.

Time	H5	H6	M5	M6	L5	L6
0 h	12,504	19,571	8582	8405	1967	1933
1 h	5245	7459	5211	5134	1745	1721
4 h	3604	4737	3809	3740	1556	1535
22 h	2100	2432	2196	2198	1210	1192
142 h	1399	1467	1366	1437	895	891

**Table 6:** Parameter values of Q-factor degradation fitting curve baking at 85 °C.

Sample	*Q*_0_	*a*	*b*	*c*	R^2^
H1	14,281	2.44 × 10^−^^4^	1.73 × 10^−^^4^	0.0552	0.97
H2	19,390	1.91 × 10^−^^4^	1.37 × 10^−^^4^	0.0452	0.95
M1	7572	2.82 × 10^−^^4^	1.49 × 10^−^^4^	0.0576	0.98
M2	7822	2.76 × 10^−^^4^	1.47 × 10^−^^4^	0.0547	0.97
L1	2118	6.00 × 10^−^^4^	1.27 × 10^−^^4^	0.0544	0.98
L2	2018	6.12 × 10^−^^4^	1.15 × 10^−^^4^	0.0517	0.97

**Table 7:** Parameter values of Q-factor degradation fitting curve baking at 105 °C.

Sample	*Q*_0_	*a*	*b*	*c*	R^2^
H3	13,048	4.96 × 10^−^^4^	4.19 × 10^−^^4^	0.0105	1.00
H4	16,388	4.18 × 10^−^^4^	3.57 × 10^−^^4^	0.0108	1.00
M3	7710	4.82 × 10^−^^4^	3.52 × 10^−^^4^	0.0118	1.00
M4	7744	4.93 × 10^−^^4^	3.64 × 10^−^^4^	0.0115	1.00
L3	2072	7.92 × 10^−^^4^	3.09 × 10^−^^4^	0.0102	1.00
L4	2061	8.06 × 10^−^^4^	3.21 × 10^−^^4^	0.0107	1.00

**Table 8:** Parameter values of Q-factor degradation fitting curve baking at 125 °C.

Sample	*Q*_0_	*a*	*b*	*c*	R^2^
H5	12,504	7.10 × 10^−^^4^	5.73 × 10^−^^4^	0.0452	0.95
H6	19,571	6.82 × 10^−^^4^	5.88 × 10^−^^4^	0.0379	0.97
M5	8582	7.34 × 10^−^^4^	5.79 × 10^−^^4^	0.0354	0.98
M6	8405	6.95 × 10^−^^4^	5.38 × 10^−^^4^	0.0393	0.97
L5	1967	1.12 × 10^−^^3^	5.80 × 10^−^^4^	0.0325	0.98
L6	1933	1.13 × 10^−^^3^	5.76 × 10^−^^4^	0.0335	0.98

**Table 9:** Test sample parameters *b* and *c* at three temperature points.

Test Temperature	*b*	*c*
85 °C	1.41 × 10^−^^4^	0.0531
105 °C	3.54 × 10^−^^4^	0.0109
125 °C	5.72 × 10^−^^4^	0.0373

**Table 10:** Data table of parameter *b* and temperature *T* at three temperature points.

*T*	In*b*
358 K	−8.8644
378 K	−7.9472
398 K	−7.4663

**Table 11:** Changes of different initial Q-factor with time during storage (T = 25 °C).

Time	*Q*_0_ = 1000	*Q*_0_ = 10,000	*Q*_0_ = 100,000	*Q*_0_ = 1,000,000
*t* = 0	1000	10,000	100,000	1,000,000
∞	982	8420	34,771	50,607

## Data Availability

Not applicable.
